# Attempt to improve functional outcomes in supracricoid laryngectomy in T2b and T3 glottic cancers

**DOI:** 10.1007/s00405-014-3244-7

**Published:** 2014-08-21

**Authors:** Małgorzata Leszczyńska, Małgorzata Wierzbicka, Maciej Tokarski, Witold Szyfter

**Affiliations:** Department of Otorhinolaryngology, Head and Neck Surgery, Poznań University of Medical Sciences, Przybyszewskiego Street 49, 60-355 Poznan, Poland

**Keywords:** Supracricoid laryngectomy, Glottic cancer, Respiration, Voice, Swallowing

## Abstract

The goal of this study was to compare the survival rate and functional outcome of an open partial horizontal laryngectomies, Type IIa and modified Type IIa (OPHL Type IIa and OPHL mType IIa), in treatment of moderately advanced glottic carcinoma. Retrospective analysis. 80 Patients underwent OPHL Type IIa and 27, OPHL modified Type IIa (OPHL mType IIa) between the years 2001 and 2009. Clinical staging was performed according to the UICC criteria (2002). Primary endpoints of study were recurrence rate, and 3- and 5-year survival time. Secondary endpoints were laryngeal functions: respiration, swallowing and voice. There were no significant differences within local and regional recurrence rates, organ preservation rate, 3- and 5-year specific disease survival rates between OPHL Type II and OPHL modified Type IIa. Significantly lower need for temporary (OPHL mType IIa 4/27, OPHL Type IIa 30/80) and permanent tracheostomy (OPHL mType IIa 2/27, OPHL Type IIa 16/80) was found. All but one patient (OPHL Type IIa) achieved unrestricted diet. Significantly differed social eating, this ability gained 25/27 OPHL mType IIa and 54/80 OPHL Type IIa (*p* < 0.05). Voice handicap index revealed a decrease in quality of life in all areas; OPHL Type IIa and OPHL mType IIa differed significantly (31 and 46 points respectively, *p* < 0.005). The MPT value (longest pitch) for OPHL Type IIa and OPHL mType IIa lasted 8 s and 10, respectively (*p* < 0.005). There was no significant difference in oncological outcomes between the two types of OPHL succeeded in the earlier extubation, thus significantly lowering the need for temporary and permanent tracheotomy and providing better long-term swallowing. Although the voice was altered in all observed OPHL patients, modified Type IIa technique proved to be superior to the Type IIa in terms of voice quality. Thus, OPHL modified Type IIa is worth promoting, as long as indications were strictly conformed.

## Introduction

Balance between morbidities: voice function, risk of persistent aspiration, higher risk for elderly and good oncological outcomes of supracricoid partial laryngectomies (SCPL) have been widely discussed in literature [1–3]. The modification of Laccourry technique of the SCPL with crico-hyoido-epiglottopexy (CHEP) was presented by Wen et al. [4], and Maoxiao and Renyu [5]. They preserved fragments of both thyroid laminas to better support the cartilage framework, to avoid stenosis and to protect swallowing function. The presented reconstruction was based on a cricothyroidopexy (CTP) in which the upper parts of thyroid laminas (not exceeding 0.5 cm) were fixed with the vicryl sutures to the cricoid cartilage, and the ventricular folds were preserved except of the region of anterior commissure (AC). This reconstructive technique resembled transglottic resection described by Calearo [6], but it differed in the amount of cartilage preservation; originally it encompassed the upper third of the laminas. The excellent oncological results of Calearo technique were presented [7], but indications for this surgery were limited to T1b glottic cancer with AC involvement.

According to a recent proposal of the European Laryngological Society (ELS) working committee on nomenclature or a systematic classification of open partial laryngectomies (OPHL), supracricoid laryngectomy with cricohyoido-epiglottopexy—CHEP, constitute Type IIa of OPHL [8]. Thus we presented the comparison of OPHL Type IIa and its modification, OPHL modified Type IIa.

The spread of a tumor from the glottis might occur either by AC invasion or by extension through the ventricles. Therefore, in tumors larger than T1b, the upper resection border should exceed the level of ventricular folds to obtain the safe margins. Since 2005, we have introduced the modified OPHL Type IIa (mType IIa) for the middle stage of glottic carcinoma and observed that functional effects of preserving even the small thyroid cartilage remnants have been beneficial for the functional outcomes. This technique is based on the laryngeal inlet resection that is as wide as that performed in Type IIa procedure and includes the mucosa and inner perichondrium to the limits of ventricular folds and the epiglottic petiole if needed, however, there is simultaneous maintenance of thyroid cartilage strips, upper laryngeal nerve, vessels and at least one crico-arytenoid unit. We assumed that conditions created during OPHL mType IIa to obtain improved neoglottis function were not contrary to oncological principles and safety. The aim of this paper is to investigate and compare the treatment outcomes of OPHL Type IIa and OPHL modified Type IIa in moderately advanced glottis cancer T2b and specific T3 cases.

## Materials and methods

To address the research purpose, the investigators designed and implemented the retrospective analysis of 107 patients with glottic cancer T2b with AC involvement. All patients were surgically treated with curative intent at the University Department of Otolaryngology, tertiary referral center and presented for evaluation and management between 01.2005 and 12.2009. The study was approved by the Poznań University Ethics Committee. The treatment protocol for the neck and the primary was based on Head and Neck NCCN version 1.2005, published in Polish literature in 2010 [9].

### Tumor staging

To be included in the study sample, patient has to have TNM stage evaluation according to the 2009 AJCC classification [10] detailed description of tumor localization in medical charts, operative reports and histological structure assessments according to the WHO classification (2005). Computed tomography to assess the primary and an ultrasound of the neck combined with FNAC (fine needle aspiration cytology) were performed if regional involvement was suspected. All patients underwent the microlaryngoscopy for biopsy and direct larynx assessment under general anesthesia. Patients were excluded as study subjects when the data were insufficient (*n* = 1), second primaries (*n* = 3) or distant metastases at the time of diagnosis were present (*n* = 1), or histology revealed the presence of a cancer other than squamous cell carcinoma (adenoid cystic carcinoma and neuroendocrine *n* = 2). Finally, all 107 patients who met the inclusion criteria had to be eligible to re-evaluation. The age of patients ranged from 30 to 73 years, mean 56.5, median 57; 12 (11.2 %) women and 95 (88.8 %) men were treated.

### Treatment

The study population was composed of the 2 cohorts of 80 and 27 consecutive patients treated by means of OPHL Type IIa or OPHL modified Type IIa (OPHL mType IIa). Medical conditions that could define patients as being selected for OPHL Type II include the extensive infiltration of the ventricle fold level (T3 ac AJCC). OPHL mType IIa was reserved for glottis cancer with extension not exceeding the ventricle (T2b). In the examined sample seven patients had their arytenoid resected. The group characteristics are shown in Table 1. Out of 19 patients undergoing elective neck dissection at the time of laryngeal primary treatment, 3 were pN1. Surgery was followed by adjuvant radiotherapy (RT) in 7 patients; 5 (8.8 %) OPHL Type IIa and 2 OPHL mType IIa (3.7 %).

**Table 1:** Patients characteristic

Primary treatment modality	OPHL Type IIa	OPHL mType IIa
No. of patients	80	27
Age mean/median	55.2/55.0	60.2/61
Women/men	10/70	2/25
Adverse findings		
Positive margins	2	2
Positive nodes (N+)	3	0
Adjuvant RT	5	2

*OPHL Type IIa* open partial horizontal laryngectomy Type IIa, *OPHL mTape IIa* open partial horizontal laryngectomy modified Type IIa

### Follow-up

The patients were routinely followed up every 6 weeks in the first 2 years and every 3 months in the 3rd–5th year. The minimal duration of a follow-up for the examined sample was 36 months.

The laryngoscopy and stroboscopy to assess the glottis was the mainstay of examination; the impact was put to exclude the local recurrence. Formation of webs, granulomas or hypertrophic mucosa was documented and patients were directed to microsurgery if they had complaints or if the larynx picture was not stable; laser procedures were performed if postoperative stenosis caused subjective dyspnoe. If local recurrence occurred, the method of salvage was analyzed with special regard to feasibility of larynx preservation.

Swallowing was assessed in 1 month and 1 year after the treatment, the Pearson-Leipzig scale [11, 12] was used. Every patient was asked two questions with the closed structure (yes or no): if they tolerate an unrestricted diet and if they can eat in social setting.

The voice handicap index (VHI) survey and maximum phonation time examination (MPT) were performed during the follow-up visit 1 year after the surgery. The VHI questionnaire assessed the impact of voice deterioration on their quality of life in functional, physical and emotional domains; using 10 questions scored 0–4 each, summed up to 120 points as maximum. The MPT value was obtained after three attempts of deep breathing with “a” phonation, the longest pitch was noted.

### End points and statistical analysis

The main predictor variable was the treatment modality OPHL Type IIa and OPHL mType IIa. Age and gender were additional predictor variables. Patients characteristics were compared by Fisher’s exact test, Chi-square test and Kruskal–Wallis test. The primary outcome variables were: local recurrence rate, 3 years local control rate, the possible salvage treatment, 3 years organ preservation rate, 3 and 5 years disease specific survival and 3 and 5 years overall survival. They were calculated using Kaplan–Meier with the log-rank test assessing equality of distributions. Secondary outcome variables were function-related and included breathing, swallowing and quality of voice. The patients were observed for early complications such as postoperative bleeding, subcutaneous emphysema, laryngeal fistula, difficulty in extubation or nasogastric tube removal and pneumonia. The late major complications in this series included dyspnea and aspiration. Student’s test was used for comparing proportions; all analyses were performed by SPSS for windows, version 15.0.

## Results

### Local control

There were 107 patients available for the 4 year assessment and 70 for the 5 year assessment. The disease control and survival outcomes with regard to treatment modality are listed in Table 2. Nine (8.4 %) patients failed primary treatment, local relapse was observed in 9 patients between 12 and 36 month of follow-up (median 16 months). Local recurrence occurred in 7 (8.75 %) OPHL Type IIa and 2 (7.40 %) OPHL mType IIa, the difference was not statistically significant (*p* > 0.05). The recurrence was found in 6/95 (6.31 %) men and in 3/12 (25 %) women, the difference was statistically significant (*p* < 0.05). 3 years local control rates for OPHL Type IIa and OPHL mType IIa were 92.50 and 85.71 %, respectively. 5-year local control rates for OPHL Type IIa and OPHL mType IIa reached 82.46 and 66.67 %, respectively. No recurrences were observed in seven patients who were administered adjuvant radiotherapy for the adverse findings (positive margins, positive nodes) during primary surgery.

**Table 2:** Oncological outcomes and local failure

Primary treatment modality	Total	OPHL Type IIa	OPHL mType IIa	*p*
No. of patients	107	80	27	
Local recurrence rate	9	7 (8.75)	2 (7.4)	>0.05
Regional recurrence rate	15 (14 %)	12 (15 %)	3 (11 %)	<0.005
3 years local control rate	63/107	92.50 %	85.71 %	0.24012
5 years local control rate^a^	47/107	82.46 %	66.67 %	0.12491
3 years organ preservation rate	63/107	92.50 %	85.71 %	0.24012
5 years organ preservation rate^a^	47/107	80 %	50 %	0.16802
3 years disease specific survival rate^a^	54/107	92.59 %	94.12 %	0.3364
5 years disease specific survival rate^a^	44/107	89.19 %	85.71 %	0.4841
3 years overall survival rate^a^	87/107	91.04 %	95.00 %	0.2074
5 years overall survival rate^a^	57/107	83.33 %	77.78 %	0.4137

*OPHL Type IIa* open partial horizontal laryngectomy Type IIa, *OPHL mType IIa* open partial horizontal laryngectomy modified Type IIa

^a^Data available for 70/107 patients

### Larynx preservation

No patients were salvaged by transoral laser or partial surgery. Total laryngectomy was indispensible in all 9 recurrences: 7 (8.75 %) OPHL Type IIa and 2 (7.40 %) OPHL mType IIa; there was no statistical difference between primary surgery modalities (*p* = 0.987) for the organ preservation rate. Salvage total laryngectomy was performed in 6/95 men and in 3/12 women, difference was statistically significant (*p* = 0.028). 3 and 5 years local control rates were equal with 3 and 5 organ preservation rates.

### Regional control

Nodal relapse was observed in 15 patients between 12 and 36 months of follow-up (median 25.6 months); neck dissection was required and metastases were confirmed in all patients. The rates for treatment modalities were: 15 % for OPHL Type IIa and 11 % for OPHL mType IIa, this difference was not significant (*p* < 0.005).

### Survival

Three patients died in preoperative period from cardiac stroke. Two patients died of regional disease in 12th and 22nd month, respectively. For the whole group 3-year disease specific survival was 92.59 % (OPHL Type IIa), 94.12 % (OPHL mType IIa), 5-year overall survival was 83.33 % (OPHL Type IIa) 77.78 % (OPHL mType IIa). Differences between the two treatment modalities were, therefore, not statistically significant.

### Postoperative complications

Early postoperative complications in the OPHL cohort included subcutaneous emphysema (12 patients), bleeding (5 patients), aspiration and pneumonia (1 patient). Wound revision was done in the first (1 patient), second (3 patients), and fifth (1 patient) day after surgery. The patient with aspiration pneumonia required antibiotics, physiotherapy and nasogastric tube maintaining for 2 weeks. There were no differences between two treatment modalities.

### Functional outcomes

Major complications in this series included dyspnea and aspiration. Specific types and incidence of functional failures according to treatment modality are presented in Table 3. A significantly lower need for temporary tracheotomy was found with OPHL mType IIa (4/27) than Type IIa (30/80) (*p* = 0.0512). Attempts to widen the airway lumen were undertaken in all these patients. Montgomery T tube was inserted in one patient after OPHL Type IIa. A laser procedure was used to widen the glottis in 28 patients: 4 OPHL mType IIa and 24 OPHL Type IIa, the difference between both treatment modalities was statistically significant (*p* = 0.1939). Sixteen patients were successfully decannulated in 3 months. A significantly lower need for permanent tracheostomy was found with OPHL mType IIa (2/27) than OPHL Type IIa (16/80) (*p* = 0.2244).

**Table 3:** Functional outcomes according to treatment modalities

Primary treatment modality	Total	OPHL Type IIa	OPHL mType IIa
No. of patients	107	80	27
Early postoperative outcome measures			
Mean day of extubation	7.598	7.750	7.148
Mean day of naso-gastric tube removal	12.604	13.038	11.333
Postoperative complications			
Bleeding	5	5	0
Pneumonia	1	1	0
Subcutaneous oedema	12	8	4
Breathing			
Laser procedure required	28	24	4
Temporary tracheotomy required (3 months)	34	30	4
Permanent tracheotomy required	18	16	2
Montgomery T tube required (6 months)	1	1	0
Swallowing			
Mean day of the naso-gastric tube removal	12.604	13.038	11.333
Successful deglutition in 1 month/1 year	49	45	4
Aspiration acc. to scale Pearson-Leipzig	94	67	1
Permanent gastrostomy required	1	1	0
Unrestricted diet	107	79	27
Social eating	79	54	25
Voice			
VHI (median score)	76	31	46
Maximum phonation time (median in s)	18.11	8	10.11

*OPHL Type IIa* open partial horizontal laryngectomy Type IIa, *OPHL mType IIa* open partial horizontal laryngectomy modified Type IIa

Swallowing abilities were assessed for six categories. The first measurement in the immediate postoperative period was the mean time until the nasogastric tube removal; in OPHL mType IIa and OPHL Type IIa, there were 11 and 13 days, respectively. Permanent gastrostomy was required in only one OPHL Type IIa case. Every patient was asked two questions (demanded answer was ‘yes’ or ‘no’): if they tolerate an unrestricted diet and if they can eat in social setting. All patients except one with gastrostomy declared that they had unrestricted diet. More troublesome was their ability to partake in social eating, which was reeducated in 25/27 OPHL mType IIa and in 54/80 OPHL Type IIa cases. The OPHL mType IIa provided better chances for undisturbed public dining (*p* = 0.0208).

The voice was analyzed in two categories. The VHI questionnaire assessing the impact of voice deterioration revealed the meaningful decrease in the life quality in functional, physical and emotional domains; with 31/120 points for OPHL Type IIa group and 46/120 points for OPHL mType IIa group. The MPT value (longest pitch), obtained after three attempts of deep breathing with “a” phonation, for both treatment modalities: OPHL Type IIA and OPHL mType IIa lasted 8 and 10.11 s, respectively, the difference was not significant (*p* < 0.005).

## Discussion

The long-term oncological and functional outcomes of T2b and selected T3 glottic cancer treated by OPHL Type IIa and its modified version OPHL mType IIa, in which the upper borders of both thyroid cartilage laminas were preserved, were investigated, compared to each other and discussed in relation to other.

### Oncological outcomes

In our series, the whole OPHL group achieved overall survival, disease specific survival, local control rate and organ preservation rate 89.71 %. Superior oncological outcomes, mainly measured by local control rates ranged in literature from 79.1 to 93.94 % and were confirmed by numerous authors [13–20] with highest score 97 % given by Laudadio et al. [21].

Treatment modalities available for T2 glottic cancer include a wide range of open surgical techniques, with vertical partial laryngectomy (VPL) or frontal anterior laryngectomy (FA) among them. Zhang et al. [22] found a significantly lower post-operative local recurrence rate in SCPL-CHEP than in VPL groups (2.6 vs. 17.8 %, *p* = 0.033). In contrast, favorable oncological results for vertical procedures presented Hartl et al. [23] with local control rate of 83 % for tumors with cartilage invasion and 94 % for those without invasion; difference was not significant, which was probably due to systematic resection of thyroid cartilage. Bakhos et al. [14] compared FA laryngectomy and CHEP, which is used more often when there was contra-lateral vocal fold spread; 5-year survival rate was 95 and 85 %, respectively, local tumor control was obtained in 87 in 83 %, respectively, but CHEP resulted in better postoperative outcomes. Wen et al. [4] presented the SCPL modalities: CTP and CHEP with recurrence rates 13 and 15 %, respectively.

Predictors of local control and survival were presented by Gallo et al. [16] in 253 samples. Univariate and multivariate analyses showed that a positive resection margin was the only important predictor of local control. A report on 291 patients by Page et al. [17] estimated the local (laryngeal) control rate to be 93.94 % and regional (cervical lymph node) control rate to be 92.05 %; in multivariate analysis, the occurrence of a second non-ENT cancer, distant metastases and margins involvement were reliable to mortality. The significantly higher frequency of local recurrence among women in OPHL, in our opinion, was related to smaller dimensions of the female larynx, compromising the efforts of the surgeon to obtain free margins.

### Functional outcomes

Early outcomes: in our study higher rates of the swallowing problems were connected with OPHL Type IIa than with mType IIa. The time to nasogastric tube removal was an important outcome measure in the early post-operative period. In the literature this varied notably from 3 to 134 days [24]. In our sample the median time of nasogastric tube removal was 12 days (11 days for OPHL mType IIa). The swallowing rehabilitation was conducted during the hospital stay, and patients were discharged when thick liquids could be consumed.

Late outcomes are dyspnea, aspiration and swallowing problems [25–27]. In our Department the planned tracheotomy was not included in our operating schedule. Temporary tracheotomy was needed in 34 patients but 14/30 in OPHL Type IIa and 2/4 in OPHL mType IIa were decannulated in time. Lucioni et al. [28] noticed that 8 % of 225 patients had laryngeal stenosis; all underwent CO2 laser one or two times, decannulation was possible in all patients except in one; authors concluded that the only reasonable contra-indication to CO2 laser could be a cranio-caudal length of the laryngeal stenotic tract longer than 1 cm. In the own 28 cases (24 OPHL Type IIa, 4 OPHL mType IIa), the CO2 laser was used to widen the neoglottis. In two females tracheotomy was needed in 2 and 6 months postoperatively but no attempts to widen the airway lumen were undertaken. Finally, 16/80 (20 %) OPHL Type IIa and 2/27 (7.4 %) OPHL mType IIa patients required permanent tracheotomy.

The compensation mechanism for swallowing after SCPL is antero-medial rotation of the remaining arytenoids that make contact with epiglottis in concordance with flexion of epiglottis, following posterior tongue movement [29, 30]. Crico-arytenoid unit preservation (cricoid cartilage and at least one mobile arytenoid cartilage) constitutes the most important condition of the effective swallowing rehabilitation. Including the arytenoids into resection margin, as in extended SCPL with both arytenoids excision, presented by Rifai et al. [31] was feasible, but active swallowing rehabilitation was principally needed. Alicandri-Ciuffiei et al. [32] sampled 106 patients and found the non-typical conformations and anomalous positioning of the epiglottis, and involvement of the lateral pharyngeal wall in the sphincteric and vibratory function of the neoglottis. On the other hand, complete epiglottis prolapse, which obstructed the neoglottis was described in three cases by Nakayama et al. [33]. In our study seven patients had one arytenoid resected; and it was combined with the transverse formation of the neoglottis and mucosal fold prolapse in five cases.

Deglutition is the main cause of morbidity in open partial laryngectomies, but the results reported in literature have been very diverse. In Farrag et al. [34], all 24 patients had a tracheostomy and percutaneous endoscopic gastrostomy (PEG) tube placement performed at the time of SCPL, with a median time to decannulation and PEG tube removal of 37 and 70 days, respectively. In 18 patients, Clayburgh et al. [35] showed similarly bad results, with an average decannulation time of 27.4 days, feeding tube removal at 87.9 days postoperatively and 67 % of patients tolerated an unrestricted diet at follow-up. Oysu et al. [36] compared the near total laryngectomy and CHEP technique; mean decannulation time was 27 and 20 days, respectively, and average nasogastric tubes removal at 23 and 17 days, respectively. In contrast to the presented above unsatisfactory results, Simonelli et al. [37] based on large sample (*n* = 116), concluded that patients with functional deglutition after SCPL showed a mild and well-tolerated degree of chronic aspiration and did not require limited oral intake. Nakayama et al. [20] observed that swallowing function (ability to eat in public) was acquired in 28/30 (93 %) irradiated and 39/43 (91 %) non-irradiated patients. Bussi et al. [38] removed the nasogastric tube in CHEP and CHP groups after an average 16 days, and good deglutition was recorded in 41 of the 44 cases (93.18 %) and adequate deglutition in the remaining 3 cases. In our series, unrestricted diet at 1 month was achieved by all of patients except one, in whom gastrostomy was needed; social eating declared 54/80 (67.5 %) CHEP and 25/27 (92.6 %) mCTP patients. The mCTP technique was shown to be valuable because it did not disturb oral intake.

### Voice

Primary RT seems to be the voice preserving treatment modality [39] but less effective oncologically. On the other hand, all manipulations in AC region worsen the glottis closure. Wen et al. [4] reported that SCPL-CTP group had significantly lower VHI scores, higher maximum phonation time and improved glottis closure than the SCPL-CHEP group. In the samples of Oysu et al. [36], the mean VHI score was 55.58 in the CHEP group and 52.78 in the near total laryngectomy group. The detailed voice outcomes of 250 SCPL patients operated on in our Department have been presented elsewhere [40]. In the present study, the quality of voice after treatment was analyzed by means of the physical subscale of the VHI and MPT examination. The VHI results differ between the examined cohorts: 31/120 in CHEP and 46/120 in mCTP were found, and MPT achieved 8 and 10.1 s, respectively. The mCTP technique was superior to CHEP in terms of voice quality.

## Conclusion

To summarize, there was no significant differences in the oncological outcomes between two surgical OPHL techniques (Type IIa and modified Type IIa). The OPHL Type IIa modification, mType IIa, was superior in earlier extubation, with significantly lower need for temporary and permanent tracheotomy, and better swallowing. Although the voice was altered in all analyzed OPHL patients, the modified Type IIa technique appeared to be superior to Type IIa in terms of voice quality. Thus, OPHL modified Type IIa is worth promoting, as long as indications for surgery were strictly confirmed.
